# Monitoring of Hadrontherapy Treatments by Means of Charged Particle Detection

**DOI:** 10.3389/fonc.2016.00177

**Published:** 2016-08-03

**Authors:** Silvia Muraro, Giuseppe Battistoni, Francesco Collamati, Erika De Lucia, Riccardo Faccini, Fernando Ferroni, Salvatore Fiore, Paola Frallicciardi, Michela Marafini, Ilaria Mattei, Silvio Morganti, Riccardo Paramatti, Luca Piersanti, Davide Pinci, Antoni Rucinski, Andrea Russomando, Alessio Sarti, Adalberto Sciubba, Elena Solfaroli-Camillocci, Marco Toppi, Giacomo Traini, Cecilia Voena, Vincenzo Patera

**Affiliations:** ^1^INFN Sezione di Milano, Milano, Italy; ^2^Laboratori Nazionali di Frascati dell’INFN, Frascati, Italy; ^3^Dipartimento di Fisica, Sapienza Università di Roma, Roma, Italy; ^4^INFN Sezione di Roma, Roma, Italy; ^5^UTTMAT, ENEA, Roma, Italy; ^6^Dipartimento di Scienze di Base e Applicate per Ingegneria, Sapienza Università di Roma, Roma, Italy; ^7^Istituto di Ricerche Cliniche Ecomedia, Empoli, Italy; ^8^Museo Storico della Fisica e Centro Studi e Ricerche “E. Fermi”, Roma, Italy

**Keywords:** hadrontherapy, real time monitoring, particle detection

## Abstract

The interaction of the incoming beam radiation with the patient body in hadrontherapy treatments produces secondary charged and neutral particles, whose detection can be used for monitoring purposes and to perform an on-line check of beam particle range. In the context of ion-therapy with active scanning, charged particles are potentially attractive since they can be easily tracked with a high efficiency, in presence of a relatively low background contamination. In order to verify the possibility of exploiting this approach for in-beam monitoring in ion-therapy, and to guide the design of specific detectors, both simulations and experimental tests are being performed with ion beams impinging on simple homogeneous tissue-like targets (PMMA). From these studies, a resolution of the order of few millimeters on the single track has been proven to be sufficient to exploit charged particle tracking for monitoring purposes, preserving the precision achievable on longitudinal shape. The results obtained so far show that the measurement of charged particles can be successfully implemented in a technology capable of monitoring both the dose profile and the position of the Bragg peak inside the target and finally lead to the design of a novel profile detector. Crucial aspects to be considered are the detector positioning, to be optimized in order to maximize the available statistics, and the capability of accounting for the multiple scattering interactions undergone by the charged fragments along their exit path from the patient body. The experimental results collected up to now are also valuable for the validation of Monte Carlo simulation software tools and their implementation in Treatment Planning Software packages.

## 1 Introduction

The use of particle therapy (PT) is becoming more and more effective for the treatment of solid cancer. The most common beams used nowadays in PT are protons, while the use of carbon ions, available worldwide only in a limited number of treatment centers, is now becoming more and more attractive.

The implementation of PT treatments that use ^4^He beams, considered so far for the treatment of uveal melanoma (1, 2) and of patients with meningioma of the skull base or spine (3) is now being considered also for pencil beam treatments (4). The use of ^16^O beams (5), another option, is also envisaged in the near future (6).

Light ion beams have a peculiar profile of released dose in tissues: this makes these beams very effective in the selective treatment of tumors, sparing the adjacent healthy tissues, compared with the standard X-ray-based treatment (7). A consequence of this higher spatial selectivity of PT is also stringent requirements on the accuracy that has to be achieved in the delivered dose monitoring.

Several factors affect the uncertainty on the position of the dose release in PT treatments. The calibration of the computed tomography (CT) images, or morphologic changes that can occur between the CT and the several irradiation sessions of a PT treatment, operated in different days, are among these possible sources of uncertainty. The correct dose release can also be affected by patient mis-positioning and organ motion during the treatment. All these contributions can sum up to a total uncertainty of the order of few millimeters on the actual voxel under treatment (8).

The treatment planning system (TPS) carefully manages the region around the tumor and the organs at risk, using a safety factor on the deliverable dose accounting for the uncertainty on its distribution. In order to protect the patient from the risks due to possible dose release misplacement, the number and the geometry of the treatment beam fields are properly designed.

A real-time monitoring procedure can, therefore, increase the quality assurance and the efficacy of a PT treatment (9). The main goal of on-line, “in-treatment,” monitoring devices is the measurement of the dose release longitudinal shape, and in particular the determination of the actual Bragg peak (BP) position for each beam energy and target voxel. The physical processes of ion beam interaction with the tissues drive the energy release to proceed through electromagnetic interactions with the patient, while the emission of radiation escaping the patient, allowing for an imaging of its source, is due to strong interactions. These processes are the basis of the development of new approaches for the determination of the BP position.

There are three nuclear processes well suited for monitoring applications: production of *β*^+^ emitters nuclei, excitation of nuclei, and charged particle production in inelastic interactions. Nuclear *β*^+^ decays produce positrons that annihilate with the electrons surrounding the emission position and yield almost back-to-back 511 keV photon pairs. Photon detection can be exploited to measure the *β*^+^ production position, and correlate it with the Bragg peak position (10–14). Since the organic tissue is mostly constituted of carbon, hydrogen, and oxygen, the *β*^+^ emitting isotopes that are most likely to be produced are ^10^C, ^11^C, ^15^O, and ^13^N.

The beam interaction with the patient body, along the path toward the target voxel, can also excite nuclei and produce de-excitations photons emitted in a very short (<1 ns) decay time interval (prompt photons). The energy range of these photons extends up to about 10 MeV (15–18).

The target nucleus fragmentation, to which the projectile fragmentation has to be added in the case of PT performed with ions heavier than protons, can result in the production of charged fragments of smaller mass that could be exploited for monitoring purposes. Such fragmentation is a high cross-section strong process that it is not trivial to describe and quantify in the energy regime of interest, where the interaction projectiles have an energy ranging between 20 and 200 MeV/u and nuclear interactions are particularly difficult to model.

The velocity of fragmentation products is close to, or even larger than that of primary ions, while the latter experience a higher stopping power. For this reason, the fragments range is longer with respect to that of beam particles: this reflects into a characteristic dose tail behind the BP. This effect is particularly relevant for the treatments; therefore, it has been studied with dedicated nuclear cross-section experiments (19, 20) and with measurements of carbon ion collisions with water targets (21–24). By these experiments, fragmentation products proven to be peaked in the forward region and mostly contained within a cone of few degrees with respect to the beam axis. Protons, which represent the largest contribution, showed instead tails at large emission angles.

Several measurements have been performed during the last decade, to evaluate the dose contribution for healthy tissues, due to the production of beam fragments. More recent studies have been focused on the possibility of exploiting the secondary particle production (and in particular the highly penetrating proton component) for monitoring purposes, as it can be used to estimate the position of the dose profile distal edge.

A first proposal was advanced in Ref. (25) that introduced the method of “interaction vertex imaging” (IVI); this method aims at reconstructing the nuclear emission vertices distribution and correlates it with the BP position, by the detection of secondary protons. In measurements performed at small angle (26, 27), using solid state tracking devices at 30° with respect to the beam direction, the distal edge of the beam has been estimated with an accuracy of 1.3 mm. In addition, variations of the beam width (transverse dimension) have been measured with a precision of 0.9 mm.

On the basis of simple geometrical considerations, the production at large angles with respect to the incoming beam direction appears to be the most interesting for monitoring applications. The quality of the single charged particle trajectory reconstruction at large angles compensates for the expected reduced statistics.

It is naturally expected that the charged particle yield at large angle remains relevant in the case of beams of particles heavier than protons. Therefore, the use of charged particle detection for the on-line monitoring of PT treatments can be especially appealing in carbon therapy. The effective implementation of this technique requires the investigation of several different aspects. The spatial distribution of charged particles emitted at large angle by a tissue-equivalent target irradiated by a therapeutic beam has to be measured accurately in order to exploit the correlation with the longitudinal dose profile and, finally, with the BP position. These measurements have to be performed as a function of different projectile types and energies, characterizing the yield of the different produced fragments and their angular distribution. Furthermore, in order to make an effective use of this approach in clinical practice, it is necessary to correlate each detected track with the position and direction of the primary beam. This also allows to take into account energy loss and scattering in the patient’s materials. Therefore, this methodology for on-line monitoring can be effectively applied to ion therapy with active beam scanning (28).

The design and implementation of a tracking device suitable for clinical applications will also require an accurate study and optimization of the detector size and positioning in order to maximize the achievable track yield and detection resolution and match the clinical requirements on the dose release monitoring.

In section 2, we will review the main available experimental results regarding the yield of charged particles produced by therapeutic beams interaction with different targets. In section 3, the methods to correlate the spatial distribution of measured secondary particles with the BP position will be presented, introducing also some general considerations about the actual feasibility of charged particle monitoring in ion beam therapy.

## 2 Charged Particles Production by Therapeutic Beams

The research and development process of novel techniques for on-line monitoring applications to PT treatments relies heavily on a detailed experimental knowledge of the secondary radiations emitted by beam interaction with the patient body.

Improving the accuracy on the measurement of the flux of secondary particles and their angular and kinetic energy spectra has been the main goal of several experiments recently performed in the research centers of Laboratori Nazionali del Sud (LNS, Catania), Helmholtzzentrum Gesellschaft für Schwerionenforschung (GSI, Darmstadt), Heidelberg Ion-Beam Therapy center (HIT, Heidelberg), and Grand Accélérateur National d’Ions Lourds (GANIL, Caen) with ion beams of different types and energies.

Helium, carbon, and oxygen ion beam interactions with water or polymethyl methacrylate (PMMA) targets of different shapes were studied, in the beam energy range relevant for hadrontherapy monitoring applications, by means of high efficiency charged particle tracking detectors.

Charged fragments, subject of this review, have been studied in two different angular ranges: particles detected at an angle *θ* with respect to the beam incoming direction between 0° and 45° (26, 27) and particles detected at large *θ* (60°, 90°, and 120°) angles (29, 30).

The *θ* spectrum of produced particles is of key importance when designing on-line monitoring devices to be integrated in hadrontherapy treatment rooms. The quest for the highest statistics data sample is hardened by the mechanical restrictions imposed by the patient positioning and related safety devices, and has also to account for beam induced background and backtracking issues for configurations at angles close to the beam incoming direction.

Although the secondary production at large angle was thought for a long time to be negligible, the experimental results have actually unveiled that the light charged fragments production, mainly protons and hydrogen isotopes, occurs even at very large *θ* angles with an integrated yield compatible with the requirements set by on-line monitoring applications.

The measurements performed with a small PMMA target (4 cm thickness) at LNS using a carbon beam with 80 MeV/u energy, also confirmed that a significant production of charged fragments occurs in BP proximity (29). This experimental result suggests that monitoring by means of charged fragments detection could be exploited also superficial tumor treatments.

Hereafter, we present the experimental setup used for the different measurements and the yield of charged particles produced at different angles.

### 2.1 Small Angle Production

A first set of measurements was performed at HIT (26), using carbon ions, of kinetic energies relevant for PT applications, impinging on a cylindrical PMMA phantom with size comparable to the human head (diameter: 160 mm, height: 90 mm). Aim of the test was to characterize the beams available in the HIT facility, looking at the secondary charged fragments produced in the interaction with the PMMA target.

The beams available in HIT have full width at half maximum (FWHM) values that are energy-dependent and range typically from 4 to 20 mm. The available energies, in the range of 48–221 MeV for protons and 89–430 MeV/u for carbon ions, correspond to beam ranges in water between 2 and 30 cm.

The directions of secondary charged particles emitted from the PMMA phantom were measured using two parallel 300-μm thick silicon pixel layers at a distance of 3.6 mm (Timepix detector). The Timepix (31) detector was placed at *θ* = 30° at a 10-cm distance from the PMMA center. The choice of the *θ* angle was driven by the needed robustness of the back-projection method for the data analysis on the one hand, and by the secondary ion yield, i.e., the multiplicity of secondary ions per primary ion, which decreases with increasing angle from the beam axis, on the other.

Different energy, width, and position configurations of the carbon beam were studied. The nominal beam intensity was set to 2 × 10^7^ ions/s and for each investigated beam parameter setting and about 2 × 10^9^ primary carbon ions were irradiated on the phantom when collecting the various data samples. The obtained secondary charged particle directions were analyzed using the back-projection method from Ref. (32).

**Figure 1:**
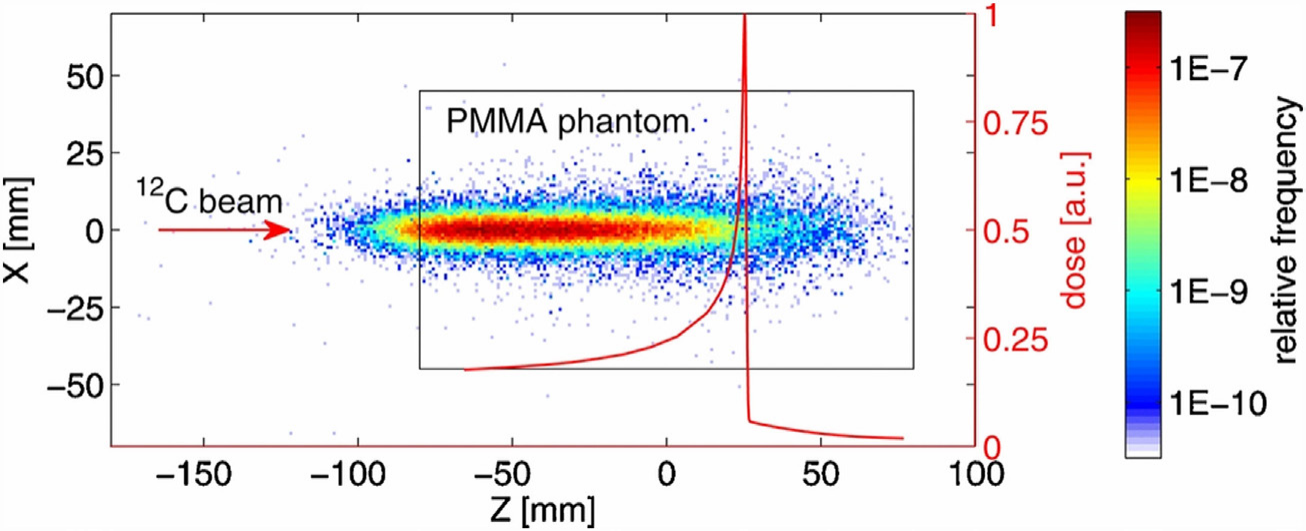
**Distribution of the track back-projections for 5 × 10^4^ measured secondary charged particles produced by the interaction of a carbon beam with a PMMA phantom at the HIT facility (26)**. The dimensions of the cylindrical PMMA phantom are illustrated by the black rectangle (view from the side). The origin of the coordinate system is aligned with the center of the phantom that was placed in the isocentre. The carbon ion beam was directed along the *Z*-axis. The depth dose distribution measured in water and scaled to the phantom water equivalent path length (WEPL) is shown by the red curve. Due to experimental limitations, the depth-dose distribution could be measured only for water depths greater than 20 mm. Initial carbon ion beam parameters: E = 250.08 MeV/u, FWHM = 4.3 mm. © Institute of Physics and Engineering in Medicine. Reproduced by permission of IOP Publishing. All rights reserved.

In all the tested configurations, a non-negligible production of charged particles was observed with a production spectrum that was correlated to the dose release in the phantom, as shown in Figure 1 for a carbon ion beam of 250.08 MeV/u kinetic energy and FWHM of 4.3 mm.

As the principal aim of the test was to characterize the beam parameters with the tracking detector, no measurement of the charged fragments flux was performed. However, taking into account that the size of the sensitive area of the used detector (~2 cm^2^) allows to cover only a small fraction (~0.15%) of the forward hemisphere around a patient, and that by decreasing the detector dead time and using a ring of detectors around the patient will greatly enhance the particles statistics, the authors concluded, on the basis of the measured yields, that the monitoring of single beam spots at the distal edge of typical brain tumor treatments with charged particles is a realistic opportunity.

Other tests were performed at small angles using PMMA and water targets: a 95 MeV/u ^12^C beam was used at GANIL (21) to study the fragments production from PMMA targets of various thicknesses at small angles; a 200 MeV/u ^12^C beam was used at GSI (22) to study the production of light fragments from the beam interaction with a 128-mm-thick water target at *θ* < 30°; a 310 MeV/u ^12^C ion beam irradiating a 21-cm-thick water target was used to study the light fragments production at 30° and 45° (27).

While the GANIL studies implemented ΔE-E telescopes at different angles, using thin silicon layers and a final stage with CsI and BGO ~7.5-cm-long scintillators placed ~20 cm away from the target, the GSI experiment used ΔE-E telescope built with a NE102 scintillator paddle followed by a BaF_2_ crystal placed 3 m away from the target. In the GANIL setup, the charged fragment identification was performed using the ΔE vs. E distributions, while in the GSI experiment performed using the 200 MeV/u carbon ion beam the additional information coming from the fragments time of flight computed using the BaF_2_ detector signals was used.

**Figure 2:**
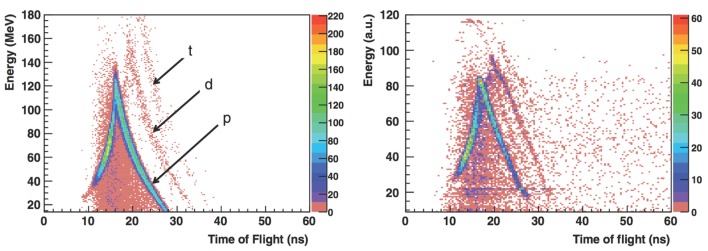
**From Ref. (27): energy vs. time of flight (expressed in nanoseconds) distributions obtained for 310 MeV/u carbon ions incident on a 21-cm-thick water target with the telescope located at 30° with respect to the beam direction at 2.2 m from the target center**. Left: simulation with Geant v.4 9.2 toolkit, right: measurements (GSI experiment). p, d, and t refer to protons, deuterons, and tritons in the simulated distributions (the background events are mainly due to fragmentation reactions in the scintillator). © Institute of Physics and Engineering in Medicine. Reproduced by permission of IOP Publishing. All rights reserved.

In the study performed with the 310 MeV/u energy carbon ion beam, a single telescope was alternatively placed at 30° and 45° with respect to the beam and in the forward direction, at a distance of 2.2 m from the target center. This telescope was composed by a thin plastic scintillator followed by a NaI(Tl) scintillator cylinder 5 cm in diameter and 5 cm in length. Thin scintillators were set upstream from the target to allow Time of Flight (ToF) measurements, triggered by incident ions, as it was done for the study performed at 200 MeV/u. The ToF of the detected particle, together with the energy deposited in the telescope detector, allowed to identify protons, deuterons, and tritons. Figure 2 shows the results obtained for the 310 MeV/u beam data sample (right) compared to Monte Carlo simulation (left) performed with the Geant v.4 9.2 toolkit (33).

High-energy particles, with ToF lower than a given threshold, escape the scintillator depositing only a fraction of their energy in it. This determines the triangular shape of the distributions for each particle type. The maximum equivalent energy deposited by protons in the 5 cm long NaI scintillator (upper point of the triangular shape distribution), on the vertical axis, was set to the corresponding energy deposition calculated by SRIM (34).

**Figure 3:**
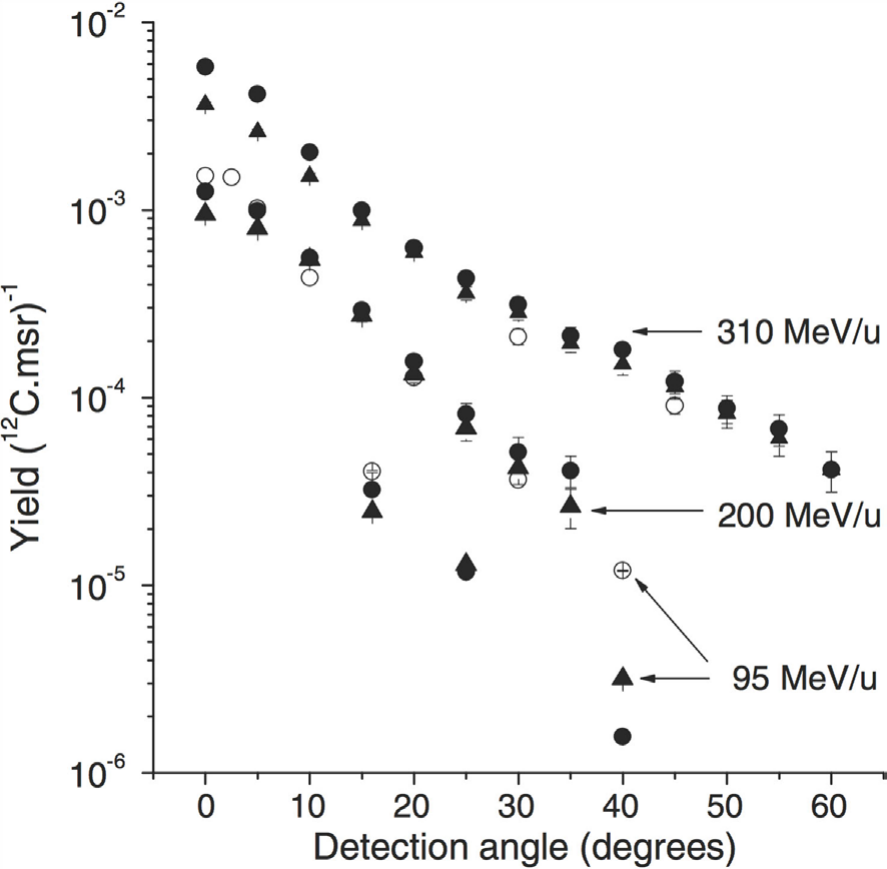
**From Ref. (27): experimental (open symbols) and simulated (filled symbols) proton emission yields as a function of emission angle and carbon ion energy (and target thickness): 310 MeV/u ^12^C, 210 mm water target (GSI experiment), 200 MeV/u ^12^C, 128 mm water target (22), and 95 MeV/u ^12^C, 25 mm PMMA target (21)**. Simulations performed with the QMD (circles) and BC (triangles) models are shown. © Institute of Physics and Engineering in Medicine. Reproduced by permission of IOP Publishing. All rights reserved.

Figure 3 shows the measured and simulated values of the detected proton yields as a function of the detection angle for the three beam energies (95, 200, and 310 MeV/u). The observed yield decrease is consistent between data and MC, as a function of the *θ* angle. The yield discrepancies between open symbols (exp. data) and filled symbols (sim.) are at most at the 40% level. The observed yields in all the angular configurations are compatible with the requirements of on-line monitoring applications, as discussed in Section 3.

### 2.2 Large Angle Production

**Figure 4:**
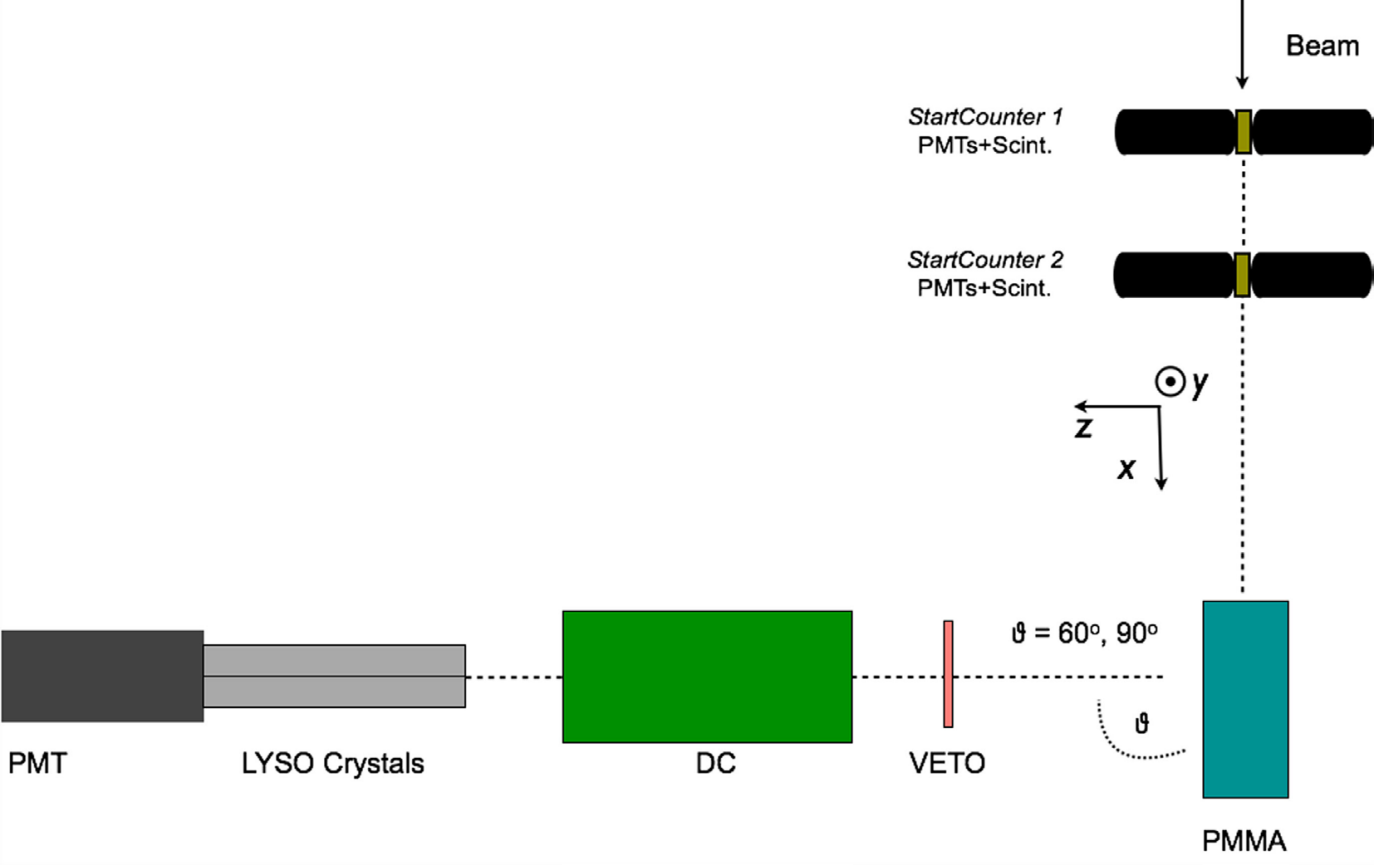
**From Ref. (30): top schematic view of the experimental setup for the 90° configuration used for the data acquisition performed in the GSI laboratory using a fully stripped carbon ion beam of 220 MeV/u**. The small differences presented in the LNS and GSI setup are described in detail in the text. © Institute of Physics and Engineering in Medicine. Reproduced by permission of IOP Publishing. All rights reserved.

The production of charged secondary particles from the irradiation of a PMMA target has been studied at large *θ* angles (≥60°) for fully stripped carbon ion beams at the LNS, GSI, and HIT facilities with energies ranging from 80 to 220 MeV/u. The experimental setup, which had only small variations in the different laboratories where the data acquisition was performed, is presented in a schematic view in Figure 4 for the experiment performed in GSI using a carbon beam of 220 MeV/u energy impinging on a PMMA target.

The tracking detectors and the details of the analysis performed on the reconstructed track sample are common to all the experiments.

An array of 4 LYSO crystals, each measuring 1.5 cm × 1.5 cm × 12 cm, was placed at 60°, 90°, and 120° with respect to the beam line, at ~70 cm from the PMMA center. The scintillation light of the crystals was detected with a PMT triggered in coincidence, within 80 ns, with the Start Counter system sketched in Figure 4. Details on the energy and time calibration of the LYSO crystals can be found in Ref. (35).

A 21-cm-long drift chamber (20) was placed at ~50 cm from the PMMA center, along the line of flight connecting the PMMA to the LYSO crystals. The drift chamber provided a 2-dimensional track reconstruction by alternated horizontal (*x*-*z* plane V-view) and vertical (*y*-*z* plane U-view) layers of wires. Twelve layers, six on each view, provided high tracking efficiency, tracking redundancy, and excellent spatial resolution, which turned out to be ≤200 μm with a single cell efficiency of ≃ 96%.

The experimental setup has been simulated by means of the FLUKA Monte Carlo software (36, 37) taking into account the trigger logic, the experimental energy thresholds and the quenching effect in the scintillator (38). MC results have been used to evaluate the setup efficiencies, geometrical acceptances, and to guide the development and tuning of the Particle IDentification.

The main difference with respect to what reported in Figure 4 for the 80 MeV/u nucleon energy measurement performed at LNS is the absence of Start Counter 2, since only one start counter was used, and the absence of the Veto detector. Furthermore, the LNS experiment was performed only detecting charged fragments at 90° with respect to the beam incoming direction.

The HIT experimental setup, used to study helium, carbon, and oxygen beams was using only one start counter, as well, but implemented a different Veto detector: a Long Thin Scintillator (LTS) was used and placed just along the PMMA, between the target and the DCH detector, in order to compute the Time of Flight of the charged fragments. The angular production configurations that were tested were, respectively, *θ* = 60° and 90°.

In the LNS experiment, the interactions of an 80 MeV/u fully stripped ^12^C ion beam with a 4 cm × 4 cm × 4 cm PMMA target were studied (29). At GSI a thicker, 20 cm × 5 cm × 5 cm, PMMA target was irradiated with a 220 MeV/u fully stripped ^12^C beam (30). During the GSI data taking the PMMA phantom was positioned on a movable table, connected to a micrometric screw, capable of shifting the phantom position along the beam (*x*) axis of about few millimeters.

The beam rate in all cases, ranged from hundreds of kilo Hertz to few Mega Hertz and was monitored with a 1.1-mm-thick scintillator (Start Counter) placed on the beam line between the beam exit window and the PMMA entrance side.

**Figure 5:**
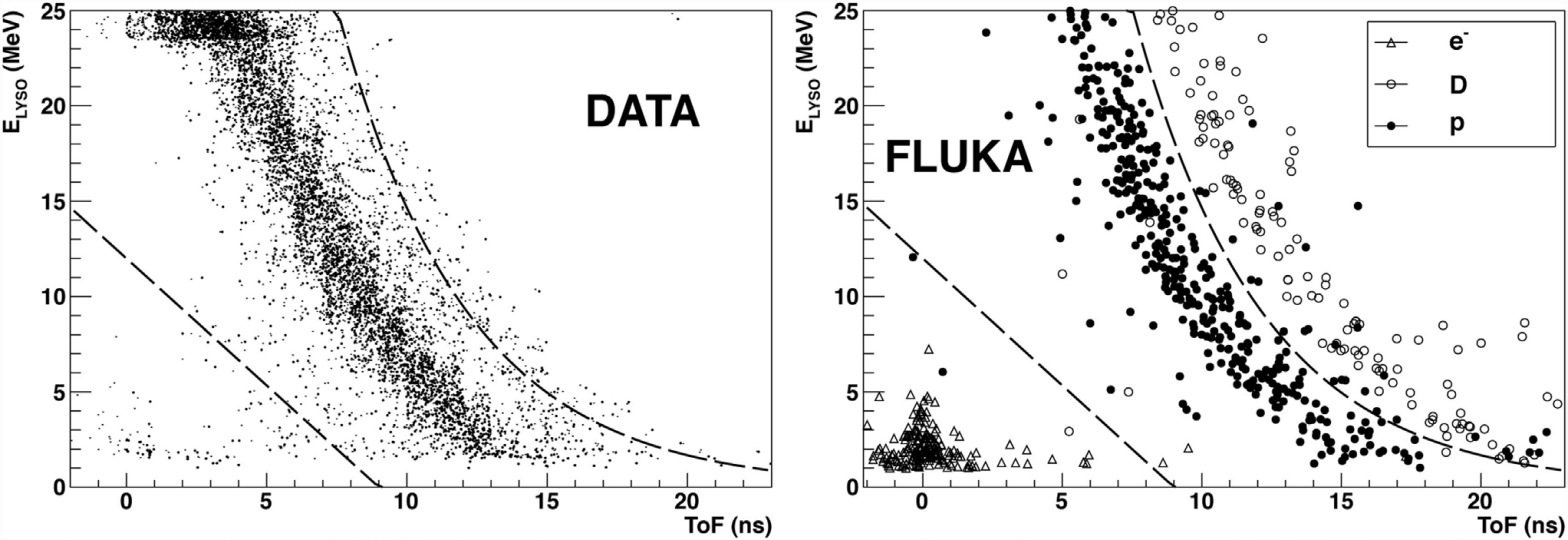
**From Ref. (29): distribution of the detected energy in the LYSO crystals as a function of the Time of Flight for the data sample collected at the LNS facility using a carbon ion beam at 80 MeV/u**. The distribution observed in the data (left) and FLUKA Simulation (right) samples are shown. © Institute of Physics and Engineering in Medicine. Reproduced by permission of IOP Publishing. All rights reserved.

Charged particles were identified starting from the tracks reconstructed in the DC using at least eight fired cells (hits), since tracks traversing the full detector are expected to have twelve hits associated. To identify the tracks, the distribution of the deposited energy in the LYSO detector (ELYSO) as a function of Time of Flight (ToF) was exploited. As an example, Figure 5 shows the measured distribution for the data collected at LNS. In the data sample (left panel) for ToF values around zero, the area delimited by the first dashed line is populated by a fast low-energy component, due to electrons produced, in the PMMA material, by Compton scattering of the de-excitation photons induced by beam interactions.

The central most populated band, delimited by the two dashed lines, is constituted by protons whose detected energy spans within a very wide range. These protons also caused the saturation of the LYSO crystals QDC for *E*_LYSO_ > 24 MeV, clearly visible. Similar populations in the (ToF, *E*_LYSO_) plane with an additional component of deuterons, above the second dashed line, are shown by the FLUKA simulation (right panel). This component is not clearly visible in data.

The data signature, with the bands relative to the different hydrogen isotopes, is common to all the experiments performed in the LNS, GSI, and HIT facilities, with a relative population of the different bands that depends on the beam type, beam energy, and settings (RMS), the PMMA thickness traversed by the fragments toward the exit window, as well as the possible different response of the LYSO crystals. The saturation that can be seen in the data distribution at energies *E*_LYSO_ larger than 23 MeV is due to the limited range of the QDC used during the data acquisition.

The only band visible in the (ToF, *E*_LYSO_) plane for the data collected at LNS (shown in Figure 5) has been defined using the data and MC distributions in order to identify and select protons in the data sample and measure their yield.

Similar distributions in the (ToF, *E*_LYSO_) plane have been observed for the GSI 220 MeV/u data both at 90° and 60°. The velocity (*β*) spectrum of secondary charged particles represents an important information for monitoring purposes, since, in order to emerge from the patient’s body and to be detected, they have to cross several centimeters of tissue. The *β* values distributions were obtained in two different ways in the LNS and GSI, HIT data analyses. While for the HIT test, the measurement of the ToF of the charged fragments was performed directly using the signal from a scintillator close to the exit path of the fragment inside the PMMA target; in the LNS and GSI setup, no dedicated detector was available and a dedicated unfolding had to be performed to take into account the travel time of the incoming ion inside the PMMA and the occurrence of secondary fragment production.

**Figure 6:**
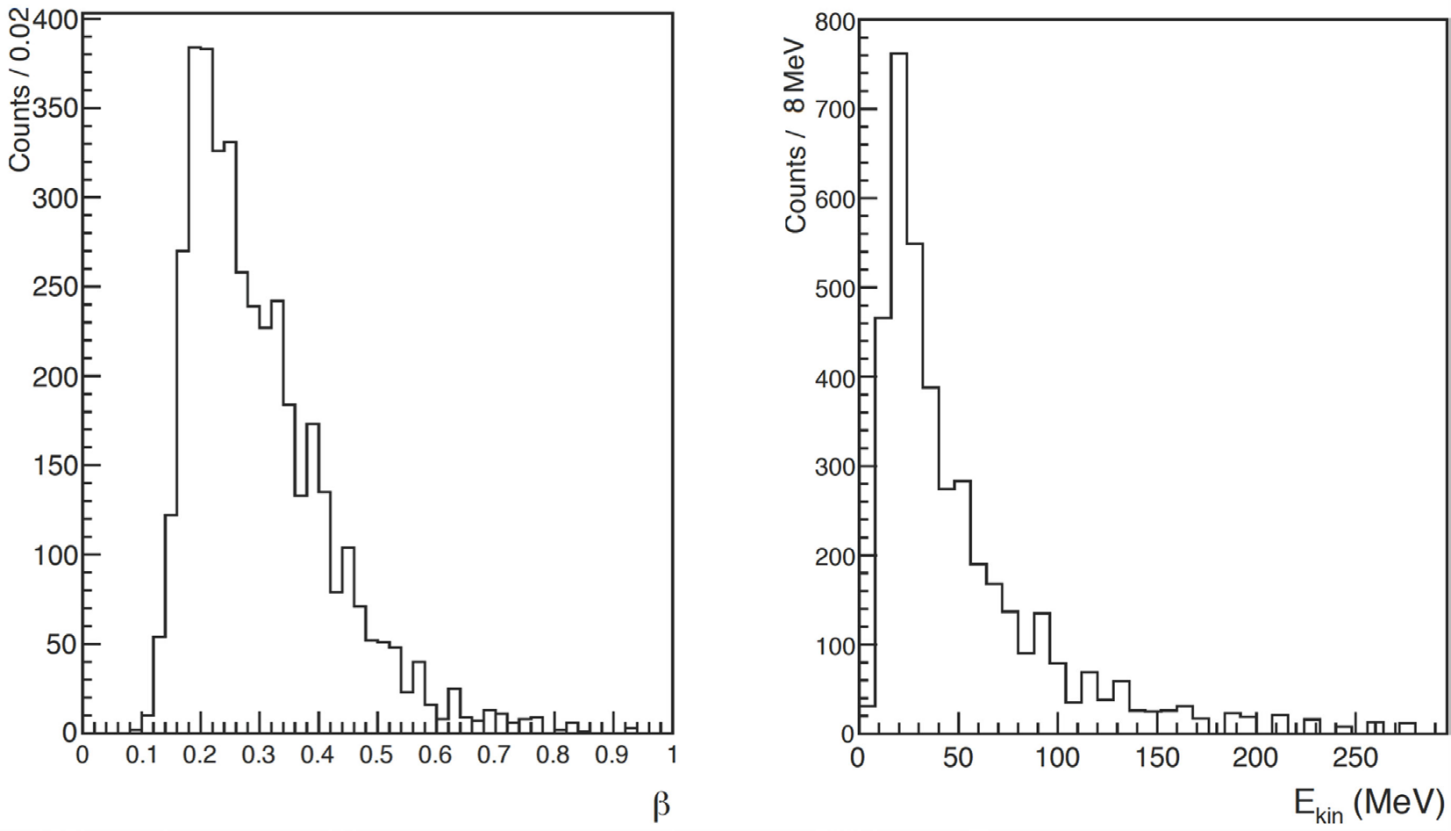
**From Ref. (29): distribution of $\beta =\frac{v}{c}$ (left) and kinetic energy (right) of charged secondary particles identified as protons in the LNS data sample**. © Institute of Physics and Engineering in Medicine. Reproduced by permission of IOP Publishing. All rights reserved.

Figure 6 shows the distributions of $\beta =\frac{v}{c}$ and the corresponding detected kinetic energy *E*_kin_ for the identified protons, obtained using the ToF measurement together with the distance between LYSO crystals and PMMA for the data collected at LNS. This detected kinetic energy can be related to the proton kinetic energy at emission time, $E_{\text{kin}}^{\text{Prod}}$, considering the energy loss in the PMMA and the quenching effect of the scintillating light for low energy protons.

In the analysis of the particle ToF for the GSI data, the finite size of the beam spot, multiple scattering of charged particles traversing the PMMA target, different energy losses, and slowing down of the various isotopes that passed through the target were taken into account. The sample is dominated by the proton contribution both in 90° and 60° samples.

**Figure 7:**
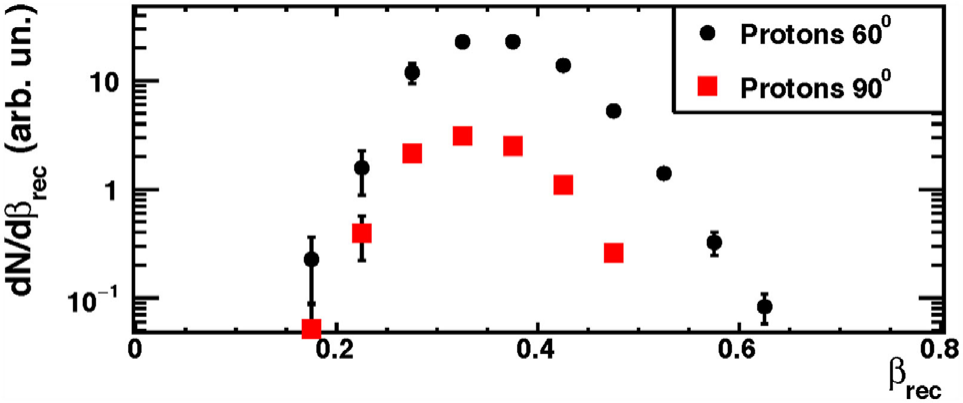
**From Ref. (30): measured emission velocity (*β_rec_*) distributions for protons in the data sample collected at the GSI facility using a 220 MeV/u carbon ion beam**. The error bars show the total (statistical plus systematic) uncertainty. © Institute of Physics and Engineering in Medicine. Reproduced by permission of IOP Publishing. All rights reserved.

The *β* distributions (*β_rec_*) of the dominant protons contribution are shown in Figure 7 for the setup configuration at 90° (squares) and 60° (circles), respectively. For each angular configuration, all spectra were normalized to the relative number of isotope species detected by the LYSO crystal. In order to use the secondary protons for monitoring purposes, the effect of crossing some centimeters of patient’s tissue has to be taken into account. Therefore, protons with detected kinetic energies greater than 50–60 MeV are the most interesting for the above-mentioned application.

Using the data collected at LNS, the flux of the secondary protons emitted from the beam interaction with the PMMA has been measured at 90° with respect to the beam direction and in the geometrical acceptance of the triggering LYSO crystals, since this configuration maximizes the sensitivity to the Bragg peak position. To determine the rate of emitted charged secondary particles that reached the LYSO crystals, the number of carbon ions impinging on the PMMA target has been calculated by counting the number of signals in the Start Counter taking into account the Start Counter efficiency, the discrimination time and the acquisition dead time.

The minimum required energy to detect a proton in the LYSO crystals was evaluated using the FLUKA simulation to be $E_{\text{kin}}^{\text{Prod}}=7.0\pm 0.5\text{ MeV}$. FLUKA has also been used to compute the emission energy $(E_{\text{kin}}^{\text{Prod}}=83\pm 5\text{ MeV)}$ of a proton with an average detected kinetic energy *E*_kin_ = 60 MeV. The uncertainty that affects the result is mainly due to the finite size of both the beam spot *𝒪* (1 cm) and profile.

The production fluxes of light charged fragments at 90° with the 80 MeV/u LNS carbon beam, obtained requiring a kinetic energy at production greater than 7 or 83 MeV are, hence, respectively (7. 1 ± 0.14_stat_ ± 0.32_sys_) × 10^−5^*sr*^−1^ and (2.14 ± 0.06_stat_ ± 0.10_sys_) × 10^−5^*sr*^−1^ with the systematic contribution mainly due to identification of protons and to the uncertainty on the production kinetic energy related to the beam’s transversal profile uncertainty. A very good stability of the result is observed with respect to the rate of the carbon ions impinging on the PMMA.

In a similar way, the results for the *Z* = 1 overall charged particles *fluxes* have been measured at GSI. The results obtained using a 220 MeV/u carbon ion beam, for the 60° and 90° experimental configurations are the following:

$$\frac{\mathrm{dN}}{{\mathrm{dN}}_{\text{C}}\mathrm{d\Omega}}\left(\theta =60^{\circ }\right)=\left(12.59\pm 0.08_{\text{stat}}\pm 0.76_{\text{sys}}\right)\times 10^{-3}{\mathrm{sr}}^{-1}$$

$$\frac{\mathrm{dN}}{{\mathrm{dN}}_{\text{C}}\mathrm{d\Omega}}\left(\theta =90^{\circ }\right)=\left(2.74\pm 0.02_{\text{stat}}\pm 0.16_{\text{sys}}\right)\times 10^{-3}{\mathrm{sr}}^{-1}$$

where the leading contributions to the systematic uncertainty is the evaluation of the dead time in data acquisition. The results are compatible with the extrapolations made from the yields measured at smaller angles and measured with different ion beam energies and target media.

**Figure 8:**
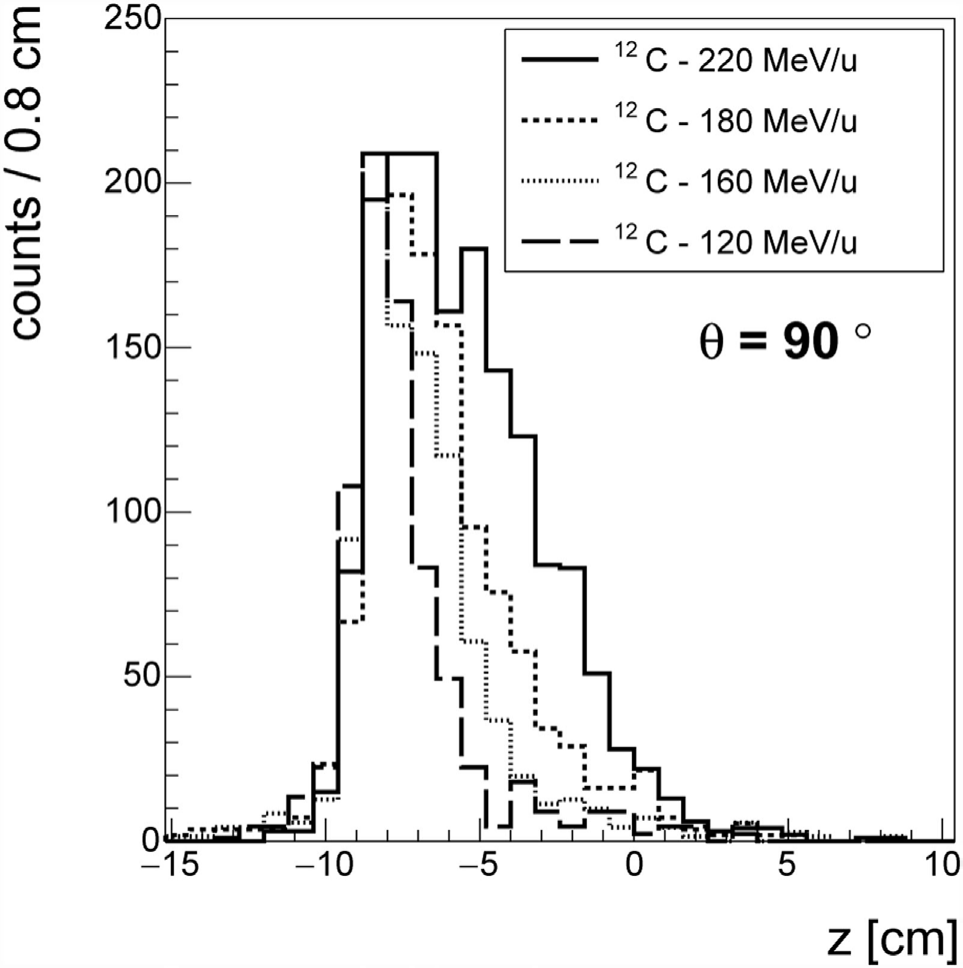
**Emission profile of the charged fragments in the case of the ^12^C beam at different energies at *θ* = 90° with respect to the primary beam direction**.

The data taken with ^12^C beam at HIT confirmed the GSI measurements and non-negligible production of protons at large angles was also observed for other ion species. Figure 8 shows the longitudinal emission profiles of protons detected at *θ* = 90°, for carbon beams at four different energies (120, 160, 180, and 220 MeV/u). As in Ref. (30), the emission shape could be correlated to the beam entrance window and the BP position. While the fluxes calculation is still being finalized, the production of charged secondary fragments at large angle is found to be consistent with what already measured in different experimental setups and centers.

The HIT experimental setup was also used to measure the secondary particles production that occurs in the PMMA targets by the interactions of ^4^He and ^16^O beams at therapeutical energies. When studying these ion beam particles, the thickness of the PMMA target was changed as a function of the ion type and energy, in order to keep the BP at about 1 cm before the end of the target. This configuration was used in order to reduce to a minimum the systematic uncertainty related to the forward interaction of the heavy fragments with the PMMA target after the BP, for the forward production studies performed with BGO detectors.

The analysis of the data collected with ^4^He and ^16^O beams is being finalized in order to produce a measurement of the absolute production fluxes: the observed raw yields are, however, encouraging for what concerns on-line monitoring applications.

## 3 The Exploitation of Charged Particle Detection for Range Monitoring

### 3.1 The Charged Particles Emission Distribution

**Figure 9:**
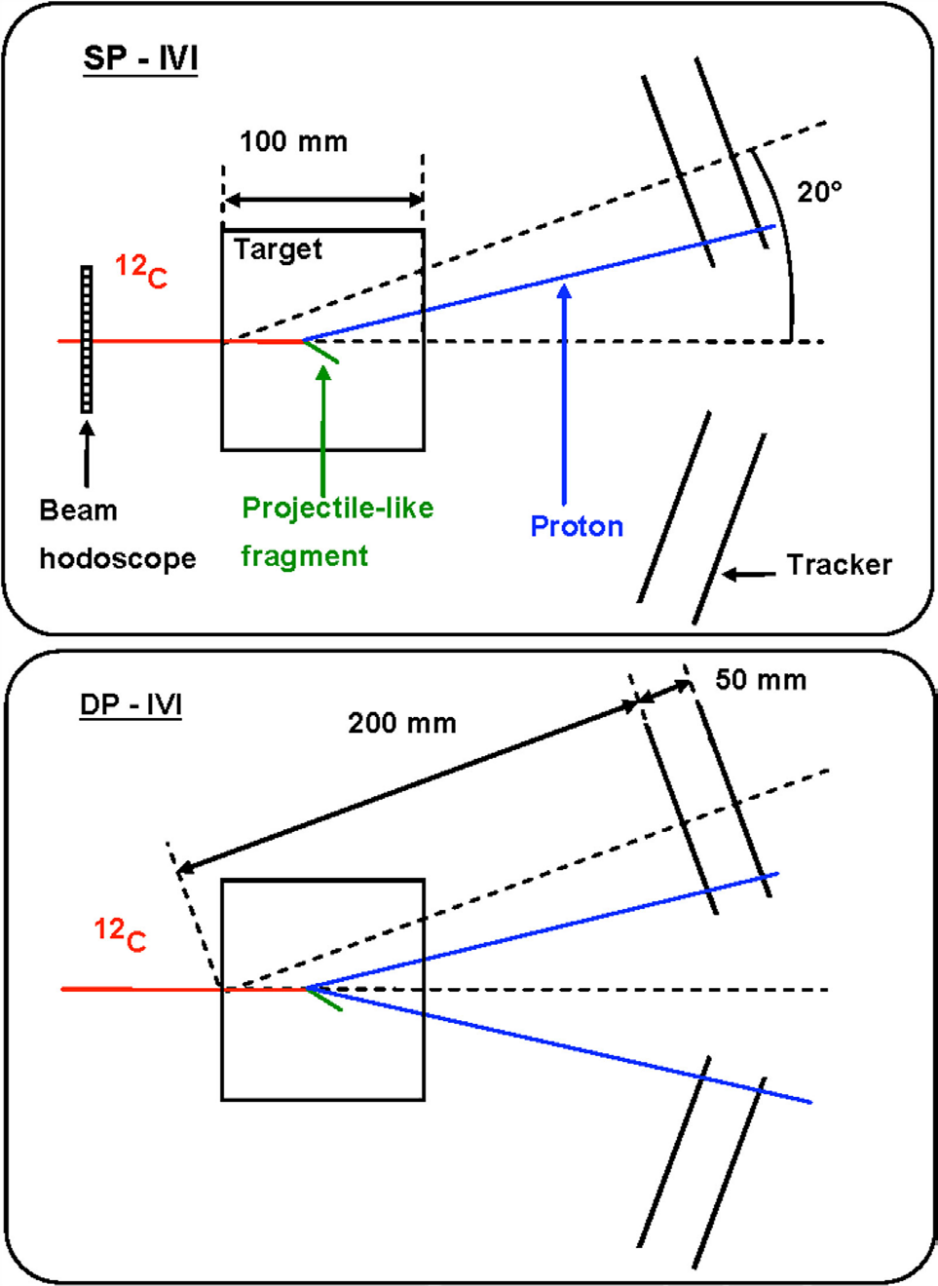
**Principle of single proton interaction vertex imaging (SP-IVI) and double proton IVI (DP-IVI) as analyzed in Ref. (27)**. © Institute of Physics and Engineering in Medicine. Reproduced by permission of IOP Publishing. All rights reserved.

The measurement of the emission shape distribution of the charged particles produced by the beam interactions with the patient tissue was recently presented in Ref. (25, 27) in the context of discussing the possible strategies for the development of an on-line tool for PT treatments monitoring. Two possible approaches were investigated with the help of Monte Carlo simulations calibrated on the measurement reported in Ref. (21, 22): single proton interaction vertex imaging (IVI) and double proton IVI, whose principle is sketched in Figure 9.

More sophisticated algorithms based on the determination of most likely trajectory [MLEM, for instance, Ref. (39)] could be also envisaged, but the simple imaging approach sketched in Figure 9 is already showing the monitoring approach fundamental principles.

Although Double Proton imaging would lead, in principle, to a safer determination of the primary path in the target by requiring the simultaneous emission and detection of a pair of protons, it reduces too much the statistic of the available signal sample.

A Single Proton imaging approach turns out then to be the only possible solution. However, in this case, the knowledge in real time of the beam position in the transverse plane during the monitoring procedure would be needed. As this information can be easily obtained from the beam delivery system, the emission point of the detected secondary particle can be obtained as the point of closest approach between the known beam line and the measured secondary particle direction.

The discussion about the optimal angle at which a monitoring detector exploiting the secondary charged radiation should be placed with respect to the primary beam direction (see Figure 9) was addressed, for the first time, in the framework of the IVI approach. Still, the choice of such geometrical parameter has a strong dependence on the angular distribution of the emitted charged secondary fragments and on the final accuracy that is achievable on the BP position. This turns out to be fundamental in any dose profile monitoring application.

At small detection angles, the emission flux increases and the charged particles energy spectrum is shifted toward higher kinetic energies. This configuration maximizes the charged fragments statistics that can escape the patient body, and be detected, while minimizing the Multiple Scattering (MS) inside the patient.

**Figure 10:**
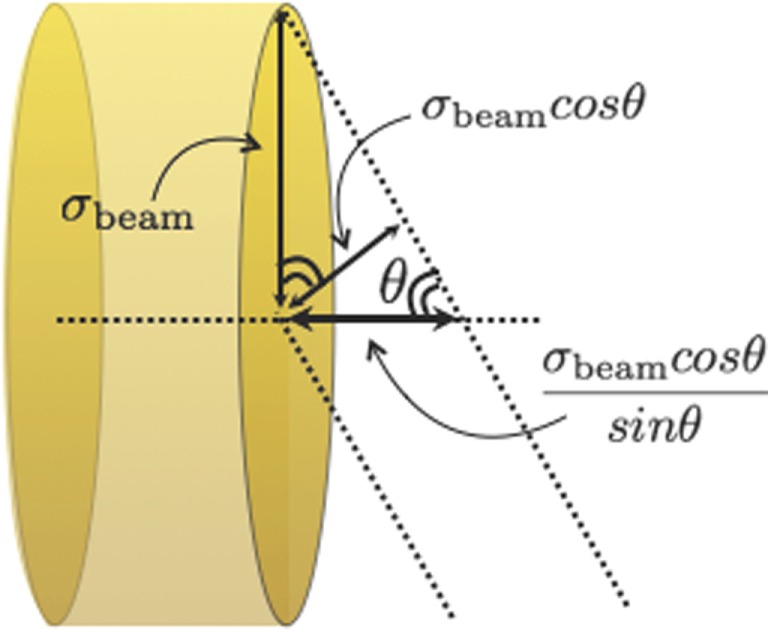
**From Ref. (30): schematic view of the beam spot size contribution to the uncertainty on the reconstruction of the fragments emission region in the case of an experimental setup placed at an angle *θ* with respect to the primary beam direction**. © Institute of Physics and Engineering in Medicine. Reproduced by permission of IOP Publishing. All rights reserved.

On the other hand, the accuracy on the charged emission point is maximal, for geometrical reasons, for orthogonal detection with respect to the beam line. As shown in Figure 10, if the projection (shadow) of the beam spot on the beam line is taken into account, the spatial resolution on the emission shape worsens as (sin *θ*)^−1^. This effect is described by a term ≃ *σ_beam_* × cot(*θ*) that becomes dominant for small detection angles. The above considerations lead the authors of Ref. (30) to focus on the measurements at large angle with respect to the primary beam directions, as already discussed in Section 2. When designing an operational setup to be used in actual treatments, the accuracy gain that could be achieved, from a geometrical point of view, at large *θ* has to be taken into account in combination with the already mentioned larger statistic and the higher average kinetic energy of the emitted particle at smaller angles, in order to obtain the necessary optimum trade off.

A key point in the range monitoring with charged particles is the correlation of the charged secondary emission profile with the beam dose release, and in particular with the BP position. A typical approach is to correlate the fall-off of the emission profile with the BP position, as shown in Figure 11 from Ref. (27), where the fall-off of the simulated SP-IVI reconstructed vertex distributions for a 95 MeV/u ^12^C beam is shown in case of different thicknesses of material (PMMA) crossed from the emission point to the detector. The smooth lines correspond to fits of equation (1) where the complementary error function (erfc) is being used.

(1) f(x)=a+b×erfc[c(x−d)]

**Figure 11:**
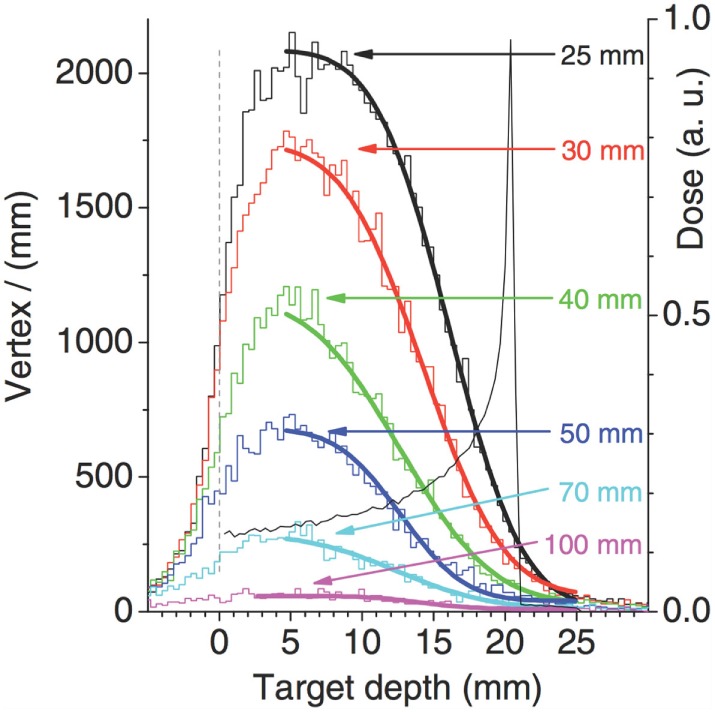
**Simulation of SP-IVI reconstructed vertex distributions for a low beam energy 12C of 95 MeV/u in PMMA for different targets thickness, as calculated in Ref. (27)**. © Institute of Physics and Engineering in Medicine. Reproduced by permission of IOP Publishing. All rights reserved.

The *d* parameter, which corresponds to the inflection point position, is assumed to provide information that can be correlated to the primary ion range.

The authors of Ref. (27) pointed out that the target thickness slightly affects the vertex distribution shape, since the secondary protons absorption affects the low-energy protons produced upstream less than those emitted at the end of the ion path. They also concluded that, probably, the proposed fit function is not appropriate in high attenuation conditions.

In Ref. (30), a different function is proposed to fit the longitudinal emission distribution of charged particles detected at large angles (equation (2)).

(2) $$f\left(x\right)=p_{0}\frac{1}{1+\exp\left(\frac{x-p_{1}}{p_{2}}\right)}\frac{1}{1+\exp\left(-\frac{x-p_{3}}{p_{4}}\right)}+p_{5}.$$

**Figure 12:**
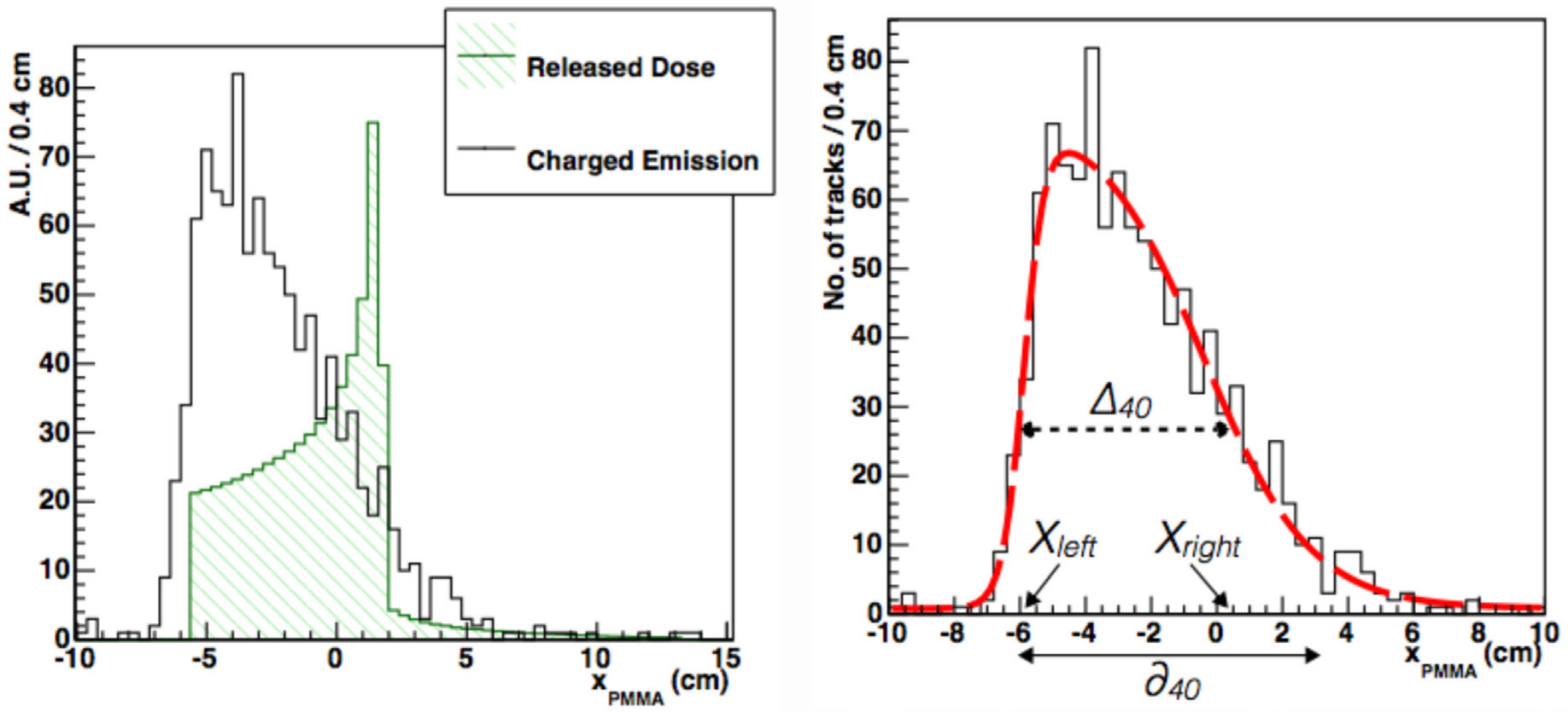
**From Ref. (30): left, longitudinal profile (solid line) of secondary charged particles as a function of the penetration in a PMMA phantom at 90° detection angle (beam entrance −6.15 cm)**. Superimposed (hatched), it is shown the beam depth-dose distribution as from MC simulations. Right, longitudinal profile (solid line) as above but with the PDF from equation (2) superimposed. The dotted and solid arrows show the graphical representation of Δ_40_ and *δ*_40_, respectively. The variables *X_left_* and *X_right_* are also shown. © Institute of Physics and Engineering in Medicine. Reproduced by permission of IOP Publishing. All rights reserved.

Figure 12 (left) shows the measured longitudinal emission distribution of charged particles emitted at 90° (solid line) by a 220 MeV/u ^12^C beam in a PMMA phantom, with a superimposed depth-dose profile as calculated with the FLUKA Monte Carlo code (36, 37) (hatched-area distribution). A clear correlation is observed between the beam entrance position in the target and the rising edge of the *x_PMMA_* distribution.

The right panel of Figure 12 shows that the *x_PMMA_* distribution is well described by equation (2): parameters *p*_3_ and *p*_1_ are, respectively, related to the rising and falling edge of the distribution, while the rising and falling slopes of the function are described by *p*_4_ and *p*_2_. A flat background contribution is accounted for through parameter *p*_5_. The beam finite size can be explicitly added at different detection angles with respect to the beam direction as a convolution term of a Gaussian function with *σ* ≃ *σ_beam_* × cot(*θ*). equation (2) accurately described all the measured emission profiles for different isotopes and data samples taken with different geometrical conditions (beam entrances) and angle configurations (60° and 90°).

Using this functional form, two quantities have been derived that are directly related to the beam range: Δ_40_ and *δ*_40_, as shown in the right panel of Figure 12. Δ_40_ represents the width of the *f*(*x*) distribution at 40% of its maximum, *X_left_* and *X_right_* being, respectively, the corresponding *x*-values at the rising and falling edges. *δ*_40_ represents the distance between *X_left_* and the *x*-intercept of the tangent to *f(x)* at *x* = *X_right_*.

Several elements influenced the accuracy of the proposed methods in monitoring the Bragg peak position: the multiple scattering undergone by the fragments inside the body, the collected sample statistics, and the intrinsic fluctuation of the emission process related to the nuclear interactions. This last contribution has been studied using a fixed number (10^3^) of detected fragments. Samples of 10^3^ tracked charged fragments were created out of the datasets acquired at each angular configuration, for a total of 13 samples at 90° and 100 samples at 60°. When comparing the measurements of Δ_40_ and *δ*_40_ performed at different angles, the finite spot size of the beam *σ_beam_* (Figure 10) was taken into account.

The accuracy on the measurement of the Δ_40_, *δ*_40_, and *X_left_* together with the average values of Δ_40_ and *δ*_40_ are shown in Table 1. The accuracy achieved for the statistic of the reference sample (1k tracks) is of the order of 3 mm. The measured absolute values of Δ_40_ and *δ*_40_ should be compared with the path Δ*_beam_* = 8.90 ± 0.03 cm, traveled by the primary beam from its entrance position in the target to the Bragg peak position, which was determined from the MC simulation used for the beam setup and calibration.

**Table 1:** **Dispersion and mean values of the parameters used to describe the charged fragments emission distribution for each angle configuration tested in Ref. (30)**.

Angle (deg)	*σ*_Δ_ (cm)	*σ_δ_* (cm)	$\sigma_{X_{\mathrm{left}}}$ (cm)	$\bar{\Delta_{40}}$ (cm)	$\bar{\delta_{40}}$ (cm)
90	0.34	0.37	0.08	6.60	9.40
60	0.31	0.28	0.09	6.83	9.44

*The parameters are correlated to the beam entrance position in the PMMA and to the BP as described in Ref. (30). The resolutions are evaluated as the RMS of the measurements performed in the different beam entrance configurations and data samples*.

The reference sample (10^3^ particles) used in Ref. (30) to validate the performances of the monitoring technique proposed, due to the reduced detector solid angle ΔΩ ≃ 10^−4^ sr, was produced by a number of carbon ions equal to ≃ 2.3 × 10^8^ at 90° and to ≃ 4.7 × 10^7^ at 60°. Those numbers can be reduced significantly (by even a factor 100) by increasing the solid angle of the tracker detector that, for clinical applications, can have a larger active area and be positioned closer to the patient.

To make a comparison with a standard carbon treatment, the number of carbon ions that are needed to give a 1 Gy dose to the distal part of the tumor (whose monitor accuracy is particularly important) has been computed: assuming that a slice of 1 cm × 1 cm with 2-mm thickness is irradiated, about 10^7^ carbon ions will be needed, distributed in a number of single spot pencil beam each one made of about 2 × 10^5^ primaries. The numbers of produced charged fragments that will be detected by a given ΔΩ detector at 90° and 60°can be easily deduced from the results quoted above.

Beside the number of primary ions that are used, another important parameter that has to be considered when discussing real case scenarios is the amount of patient tissue crossed by the secondary particles in their exit path, before their detection. As the absorption increases with the traversed matter, a reduction of the flux up to a factor 10 has to be considered in case of tumors that are located very deeply in the patient body, as can be inferred from Figure 11.

The accuracy achievable is, therefore, function of the signal tracks statistics, and hence on the dose administered in a given fraction, and of the absorption due to the depth of the tumor. In order to enhance the signal statistics, a possible strategy is to envisage the monitor of a group of pencil beams in the same treatment slice. At the same time, the maximization of the geometrical acceptance of the monitor device is also crucial, getting as close as possible to the patient, to enhance the collected tracks sample statistics and, hence, the accuracy attainable with a small number of pencil beams.

### 3.2 An Application to the Clinical Environment: The Dose Profiler

As discussed in the previous section, in order to exploit the detection of charged particles for range monitoring in PT a large acceptance is needed. Other requirements to be taken into account in the detector design are compactness, reliability, and high tracking efficiency. We will consider, as practical example to discuss the application to a clinical environment, the Dose Profiler (DP) device (40) developed in the framework of the INSIDE (INnovative Solutions for In-beam Dosimetry in hadronthErapy) project (41). The tracker implemented within INSIDE is built out of six double planes of scintillating fibers oriented in two orthogonal views to provide bi-dimensional readout, with a sensitive area of about 20 cm × 20 cm. The fiber transverse section (500 μm × 500 μm) provides the necessary spatial resolution for an accurate reconstruction of the charged tracks, considering that the resolution on the fragment emission point is dominated by the Coulomb and nuclear scattering undergone in the patient tissues in the exit path.

Some of the practical features related to the application of a charged particle-based monitoring technique to PT treatments were addressed using Monte Carlo simulations. A real case scenario was studied in detail by performing an accurate FLUKA MC simulation of the treatment with ^12^C ions undergone by a patient at the Italian hadrontherapy center CNAO (42). The treatment was a two-port irradiation of a chordoma (volume of about 45 cm^3^) placed almost at the center of the head.

**Figure 13:**
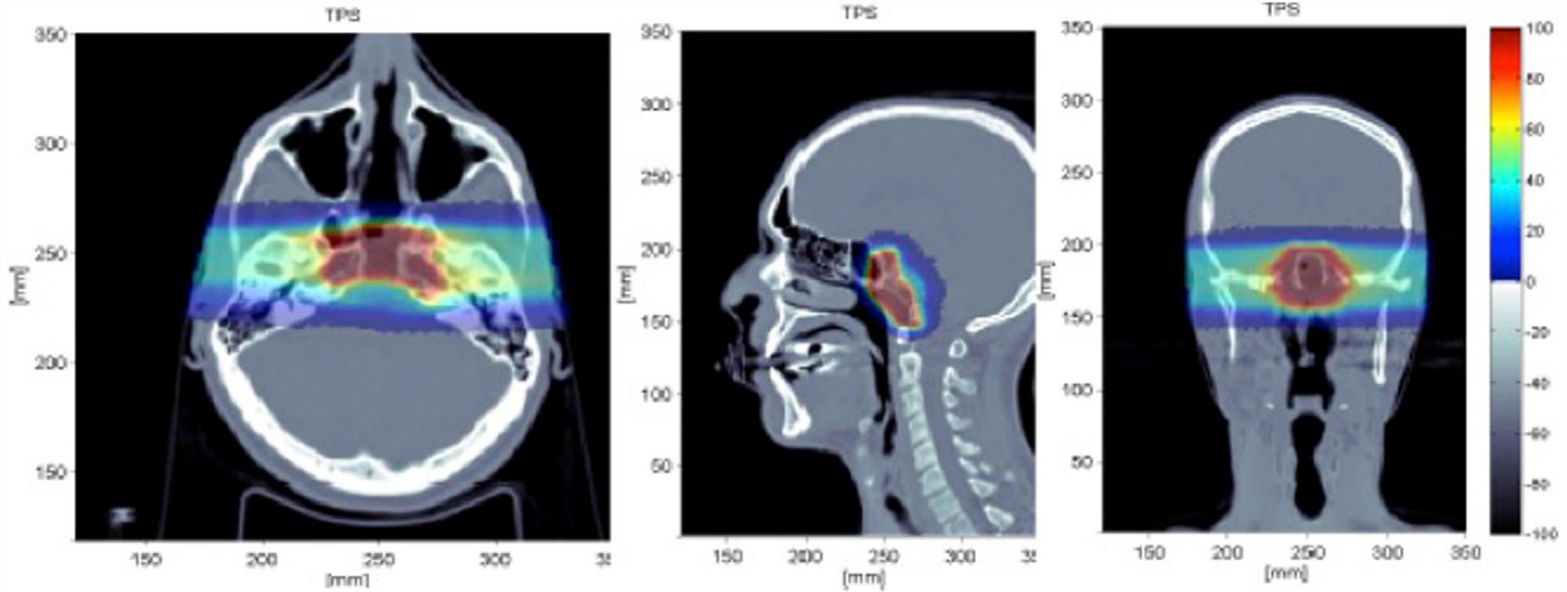
**Simulated treatment plan of a chordoma as displayed by the Treatment Planning System (Syngo TPS by Siemens) for a patient treated with ^12^C ions at the Italian hadrontherapy center CNAO (42): transaxial (left), sagittal (center), coronal (right) views**. Courtesy of CNAO.

The simulation reproduced all the details of the beam delivery and the actual geometry of the patient, importing the CT image (see Figure 13). The output from the Treatment Planning System (Syngo by Siemens) was coupled to the simulation input and a single fraction of the treatment was considered for one of the two beam ports. The energy of the ^12^C primary ions for such a treatment was in the range of 137.28–243.42 MeV/u. The total number of primaries used for the simulation of a given treatment fraction was 2.7 × 10^8^.

Prompt photons and secondary protons emerging from the patient with an energy greater than 1 and 20 MeV, respectively, were studied. The INSIDE tracking detector was placed at a distance of 40 cm from the tumor at about 60° with respect to the beam direction. The total number of photons and protons entering in the detector acceptance are 2.7 × 10^6^ and 6.4 × 10^5^, respectively.

**Figure 14:**
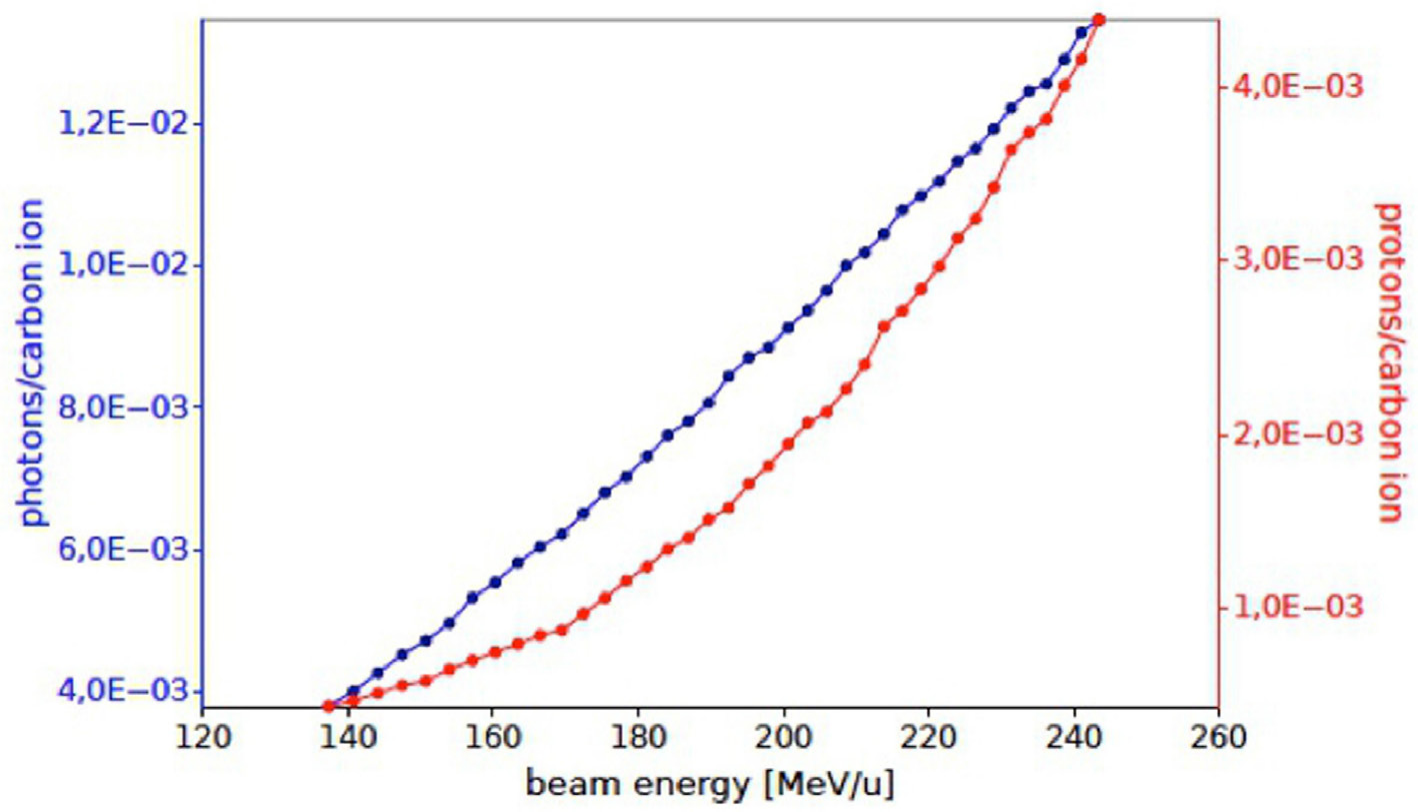
**Number of prompt photons (blue) and protons (red) per carbon ion in the acceptance of the INSIDE Dose Profiler detector as obtained by the simulated treatment planning at an angle of ~60° with respect to the primary beam, for a single fraction of the treatment of Figure 13**.

In Figure 14, the expected numbers of photons and protons for each carbon ion entering the detector active area, placed at 60° with respect to the primary beam incoming direction, are shown as a function of the beam energy. The Monte Carlo evaluation of the proton flux at 220 MeV/u is compatible with the results reported in Section 2.

The application of these techniques to the “online” (in-treatment) monitoring of the beam range requires a calibration of the measured parameters used to describe the longitudinal emission distribution (the Δ_40_, *δ*_40_ parameters introduced in the previous paragraph). The dependence of Δ_40_ and *δ*_40_ against the actual BP position for the energy of interest or, correspondingly for the carbon beam range of interest in PT, has to be performed by means of an extended campaign of experimental measurements.

In order to implement the monitoring technique here proposed in actual clinical cases, a possible strategy is described in the following. Any complex geometry, like the case of a patient, having different materials, densities, and thicknesses will produce a longitudinal emission profile that will be quite different from the reference case presented so far. However, since all the relevant information is in principle contained in the patient’s CT, it is possible to develop a method that allows to take into account all the deformations of the secondary charged emission shape due to the absorption of charged fragments in the patient tissue, as indicated in Figure 11.

**Figure 15:**
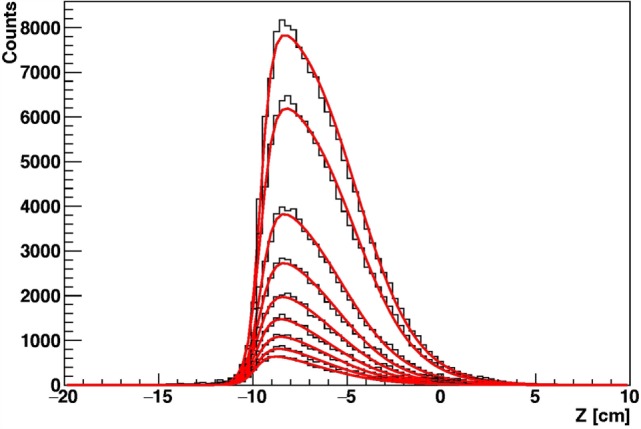
**Simulation of the reconstructed longitudinal profile of the emission points of secondary protons as detected at 90° with respect to the beam direction, for ^12^C beam of 220 MeV/u irradiating a cylindrical PMMA target, for different targets radii**.

The reference emission shape, whose correlation with the BP position is known, can be obtained from the measured emission shape by unfolding the expected absorption as a function of thickness (obtainable from the CT) along the reconstructed track direction. A function describing particle absorption in different materials can be reliably obtained by Monte Carlo simulation. In order to give a proof of principle of the proposed method, we have developed a Monte Carlo simulation calibrated with the data reported in Section 2. Using the same beam and detector conditions employed in the real case scenario simulation shown in Figure 13 (primary ^12^C beam of 220 MeV/u, DP detector), the attenuation of protons emitted at 90° with respect to the beam incoming direction has been obtained for PMMA as a function of the thickness of material crossed by the fragments. Results are shown in Figure 15.

**Figure 16:**
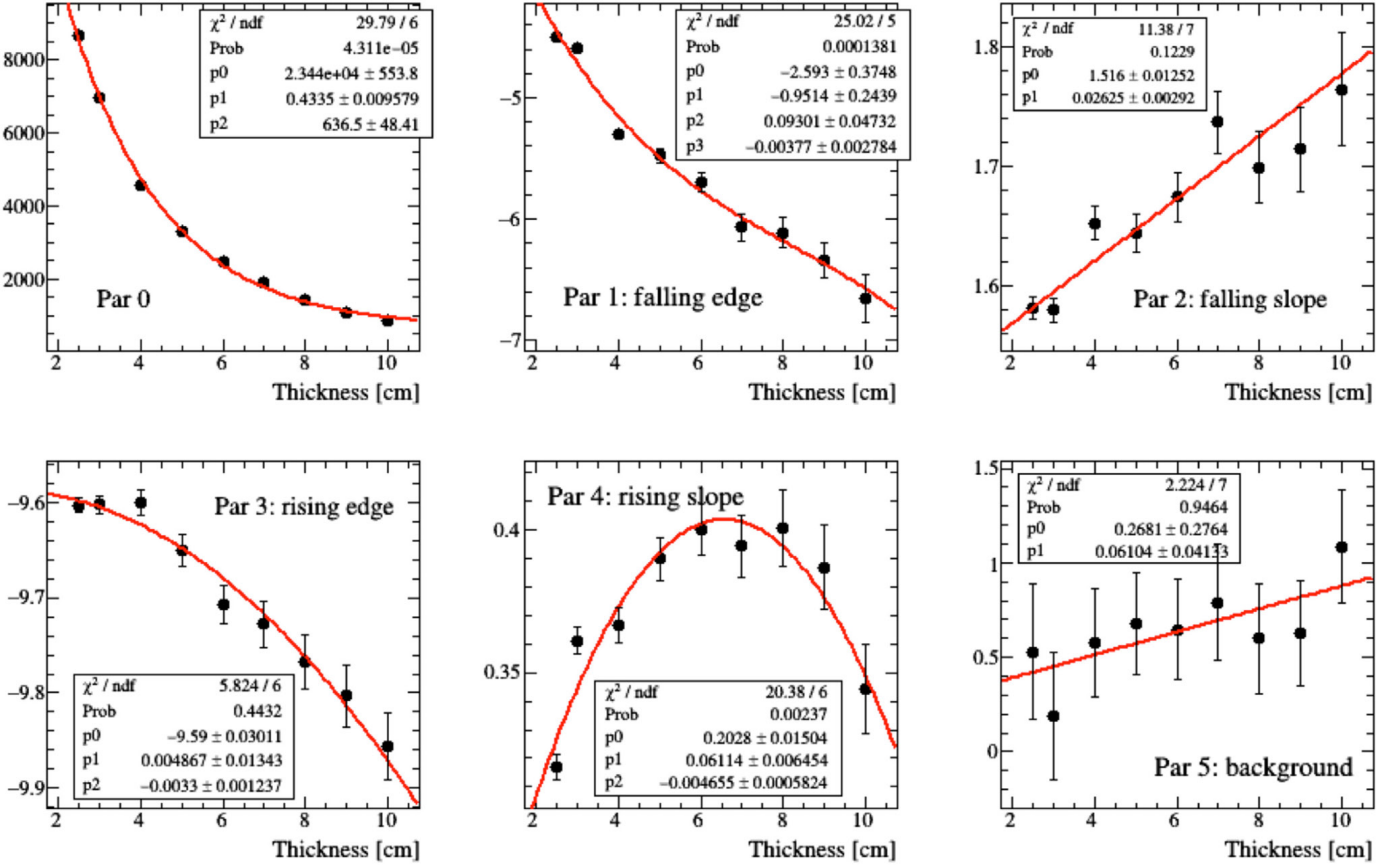
**Polynomial fit modeling the evolution of the parameters of equation (2) resulting for different thicknesses of material crossed by the charged secondary particle as shown in Figure 15**.

In real case scenarios, look-up tables will be used for different beam energies and “water equivalent material” thicknesses. The emission shapes predicted for different thicknesses of Figure 15 have been fitted using the function of equation (2). In order to parameterize the functional shape for an arbitrary value *x* of thickness, the variation of the six *p_i_* parameters that enter the function definition has been studied as a function of *x* by means of simple polynomial fits, as shown in Figure 16, in the 2.5–10 cm range.

Once the “look-up tables” are available, to take into account the variation of the *p_i_* parameters as a function of the material thickness, the emission function of equation (2) can be generalized as a two variables function of *z*, the emission point along the beam path, and of the crossed material thickness *x* traversed in the escape path from the phantom, using the *p_i_*(*x*) functions:

(3) $$f\left(z,x\right)=p_{0}\left(x\right)\frac{1}{1+\exp\left(\frac{z-p_{1}(x)}{p_{2}(x)}\right)}\frac{1}{1+\exp\left(-\frac{z-p_{3}(x)}{p_{4}(x)}\right)}+p_{5}\left(x\right).$$

A weighting function can be defined for each charged secondary track with emission point reconstructed at position *z* and with crossed material *x* before escaping the patient:

(4) $$w\left(z,x\right)=\frac{f(z,x_{0})}{f(z,x)}$$

Here, the reference *x*_0_ correspond to the minimum 2.5 cm thickness of the PMMA used to collect the data (30) on which the simulation has been trained. In order to take into account the absorption effect, any detected track will contribute to the emission shape with a weight *w*(*z, x*) evaluated using the measured *z* and the *x* obtained from CT.

**Figure 17:**
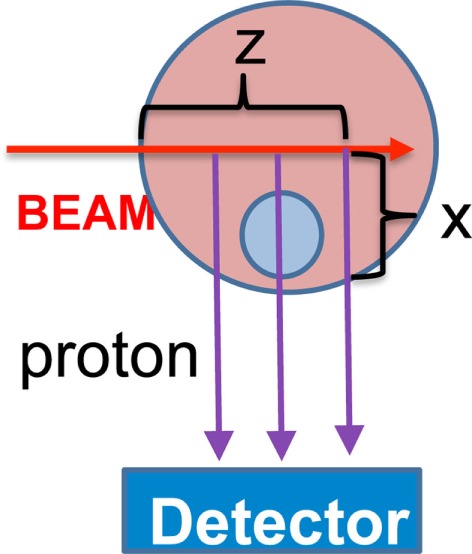
**Simulation setup for a proof of concept of the material absorption deconvolution**.

In order to demonstrate the feasibility of the proposed approach, we have simulated a simple system, shown in Figure 17, where a ^12^C beam propagates in a PMMA sphere of 10-cm radius (density *ρ* = 1.2 g/cm^3^), that contains a smaller sphere of density *ρ_o_* = 0.6 g/cm^3^ and radius = 3 cm. The detector used for the MC simulations is the INSIDE Dose Profiler, placed at a 40 cm distance from the center of the larger sphere. In this case, the thickness *x* of crossed material can be calculated analytically.

**Figure 18:**
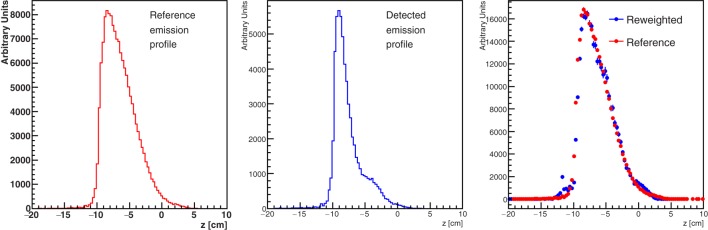
**True (left panel) and detected (middle panel) secondary charged particles emission profiles obtained from the MC simulation setup of Figure 17**. The right panel shows the effect of the re-weighting procedure described in the text, needed to account for the different material traversed by the secondary fragments.

Figure 18 shows the result of the unfolding procedure. The left panel shows the MC profile of the emitted charged secondary particles as produced by the beam, while the emission profile reconstructed by the detector is shown in the central panel. The distortion in the reconstructed shape due to the different material thickness is evident as well as the heavy implications for the correct evaluation of the BP position when using the biased reconstructed distribution without any correction. By weighting each reconstructed track with the inverse of the weight *w*(*z, x*) defined in equation (4), the result shown in the right panel is produced, where the re-weighted profile is superimposed to the generated one. The nice agreement obtained proves the feasibility of a measurement of the true charged secondaries emission profile, once the detailed map of the material crossed by the detected protons is known. In a real case scenario, a software system capable of exploiting on-line all the useful information from the CT has to be implemented.

The proposed technique, beside the monitoring of the BP position, could also be used to provide additional information about the patient positioning. By the correlation of the beam entrance position in the patient to *X_left_* (for the definition and the expected resolution refer to Table 1 and Figure 12) a fast and precise feedback on possible patient mis-positioning could be provided during the treatment.

## 4 Concluding Remarks

Nowadays, the baseline approach for PT range monitoring is through PET imaging, typically undergone by the patient immediately after the treatment. In order to improve the treatment reliability and ensure an accurate control on the dose deposition, different research groups are developing and optimizing a dedicated monitoring device capable of being operated during the treatment.

Techniques based on the detection of secondary prompt photons are recently starting clinical experimentation: first prototypes are being developed and tested “in room” with an optimization focused mainly on applications to proton therapy (43). At the same time, a monitoring technique based on the detection of charged particles is being developed. The preliminary studies and experimental results presented in this review showed that promising performances are expected for such technique when applied to the monitoring of ion treatments, as proton projectiles would produce an insufficient yield of charged secondaries.

An advantageous strategy that can be pursued to achieve the desired monitoring space resolution implies the detection of fragments emitted at large angles with respect to the beam incoming direction, even at the price of having a lower yield of particles as they are emitted preferentially in the forward direction. In this case, the reduction of the MS undergone inside the patient body and the reduction of the beam shadow effect will help significantly in matching the monitoring requirements posed by the clinical application.

The application of a charged particle-based monitoring could be problematic in case of deep seated tumors, because of the re-absorption of charged secondaries inside the patient itself. However the technique feasibility is fully recovered in the context of hypo-fractionated treatments. For those treatments, the need for *on-line* range check is even more compelling as very large doses are delivered in one or few shots, and the total dose for the single irradiation session, and the related secondary yields, can be almost one order of magnitude larger than the standard treatments.

The three leading techniques that are nowadays being considered for in-beam range monitoring (PET, prompt gammas and charged particles) offer in principle different advantages and pose different problems. The performance comparison of the three approaches is not trivial. One reason is that many of the proposed detectors and approaches still do not have firmly established performances, since they are in a research and development phase. Another reason resides in the limited reliability of the nuclear interactions description in Monte Carlo codes in the energy range of few hundreds MeV/u. In this respect, the process of secondary charged particles emission at large angles is one of the most difficult to benchmark for the existing models.

The increasing amount of data coming from dedicated experimental campaigns, and the impressive modeling activity performed by the code developers, is allowing the MC simulation research field to evolve quickly. An example of recently achieved results is in Ref. (44) and in Ref. (13) specifically for the PET technique.

Finally, the relative performances of the three techniques strongly depend on the tumor size and position and on the absolute dose release foreseen for a given treatment. Combined approaches in which two or more secondary signals are simultaneously exploited are, thus, promising.

A first example of such integrated approach is being developed within the INSIDE (41) project: here, two planar PET heads made of pixellated LYSO crystals are operated in combination with the Dose Profiler, a large area charged particles tracker made of orthogonal layers of scintillating fibers. The PET subsystem has ToF and DAQ capabilities that allow for in-beam operation, while the tracker is focused on the detection of charged secondaries emitted at large angle (60°–90°) with respect to the beam direction. The test of the integrated device is foreseen in 2016, in the CNAO therapy center.

## Author Contributions

SM: Monte Carlo simulation and the reconstruction work reported in Section 3.2. GB: data taking, data analysis, and Monte Carlo simulation. FC: data taking and Monte Carlo simulation. EL: data taking and data analysis. RF: experiment management and data analysis. FF: data analysis. SF: experiment preparation. PF: data taking and data analysis. MM: experiment preparation, data taking, and data analysis. IM: experiment preparation, data taking, and data analysis. S. Morganti: data taking. RP: data analysis. LP: experiment preparation and data taking. VP: project coordinator, data analysis, and Monte Carlo simulation. DP: experiment preparation and data taking. A. Rucinski: data analysis. A. Russomando: data taking. A. Sarti: experiment preparation, data taking, and data analysis. A. Sciubba: experiment preparation and data taking. ES-C: laboratory activity and data taking. MT: experiment preparation, data taking, and data analysis. GT: data analysis and laboratory activity. CV: data analysis.

## Conflict of Interest Statement

The authors declare that the research was conducted in the absence of any commercial or financial relationships that could be construed as a potential conflict of interest.
